# The development of type VI glandular trichomes in the cultivated tomato *Solanum lycopersicum* and a related wild species *S. habrochaites*

**DOI:** 10.1186/s12870-015-0678-z

**Published:** 2015-12-12

**Authors:** Nick Bergau, Stefan Bennewitz, Frank Syrowatka, Gerd Hause, Alain Tissier

**Affiliations:** Department of Cell and Metabolic Biology, Leibniz-Institute of Plant Biochemistry, Weinberg 3, 06120 Halle, Saale Germany; Interdisciplinary Center for Material Sciences, Martin-Luther University Halle-Wittenberg, Heinrich-Damerow-Str. 4, 06120 Halle, Saale Germany; Biocenter of the Martin-Luther University Halle-Wittenberg, Weinbergweg 22, 06120 Halle, Saale Germany

**Keywords:** Development, Electron and fluorescence microscopy, Immuno-labelling, *Solanum habrochaites*, *Solanum lycopersicum*, Tomato, Type VI trichomes

## Abstract

**Background:**

Type VI glandular trichomes represent the most abundant trichome type on leaves and stems of tomato plants and significantly contribute to herbivore resistance, particularly in the wild species. Despite this, their development has been poorly studied so far. The goal of this study is to fill this gap. Using a variety of cell imaging techniques, a detailed record of the anatomy and developmental stages of type VI trichomes in the cultivated tomato (*Solanum lycopersicum*) and in a related wild species (*S. habrochaites*) is provided.

**Results:**

In both species, the development of these structures follows a highly reproducible cell division pattern. The two species differ in the shape of the trichome head which is round in *S. habrochaites* and like a four-leaf clover in *S. lycopersicum*, correlating with the presence of a large intercellular cavity in *S. habrochaites* where the produced metabolites accumulate. In both species, the junction between the intermediate cell and the four glandular cells constitute a breaking point facilitating the decapitation of the trichome and thereby the quick release of the metabolites. A strongly auto-fluorescent compound transiently accumulates in the early stages of development suggesting a potential role in the differentiation process. Finally, immuno-labelling with antibodies recognizing specific cell wall components indicate a key role of pectin and arabinogalactan components in the differentiation of type VI trichomes.

**Conclusions:**

Our observations explain the adaptive morphologies of type VI trichomes for metabolite storage and release and provide a framework for further studies of these important metabolic cellular factories. This is required to better exploit their potential, in particular for the breeding of pest resistance in tomato.

**Electronic supplementary material:**

The online version of this article (doi:10.1186/s12870-015-0678-z) contains supplementary material, which is available to authorized users.

## Background

The plant epidermis is an essential tissue which plays critical roles not only in the interaction of plants with their biotic and abiotic environment, but also in development and the acquisition of nutrients. These diverse functions are fulfilled by a range of epidermal differentiations, from root hairs underground to stomata and different sorts of trichomes in the aerial parts. Trichomes are epidermal protuberances, which can assume an enormous diversity of shapes and sizes [1, 2]. They can be uni- or multicellular, glandular or non-glandular, and within a plant species distinct trichome types can be present. In contrast to *Arabidopsis thaliana* which has essentially a single type of unicellular non-glandular trichomes, species like tomato can display up to seven different types with no less than four different types of glandular trichomes [3]. Trichomes make an attractive system to study fundamental processes of organ development and differentiation because they are not essential organs. Hence, the trichomes of *Arabidopsis thaliana* have been the object of numerous genetic and molecular studies, leading to a detailed dissection of the molecular genetics of their development and patterning processes [4]. In comparison, there are until now comparatively few molecular genetic studies on the development of glandular trichomes. Recently we have proposed that tomato (*Solanum lycopersicum* and related wild species) serves as a model system for the research on glandular trichomes, due to its extensive genetics resources, a sequenced genome and an active research community [2]. Among the glandular trichomes of tomato, three major types can be distinguished. Type VII are short glandular trichomes with a single stalk cell and a berry-like head with a variable number of secretory cells. In tobacco, short glandular trichomes that resemble the tomato type VII trichomes produce proteins called phylloplanins, which display antifungal activity [5]. Type I and type IV trichomes are related and are of the capitate type, with a multicellular stalk and one to several glandular head cells. Type I trichomes are long and present in several tomato species including *S. lycopersicum*, while the shorter type IV are restricted to wild species such as *S. pennellii* and *S. habrochaites*. There is now substantial evidence that type I and type IV trichomes are the main site of synthesis of a variety of acyl sugars [6, 7]. Type VI trichomes represent the most abundant trichome type in several tomato species, including *S. lycopersicum*, and have a specific architecture with four glandular cells arranged on one plane atop one intermediate cell and a single stalk cell. They are the site of biosynthesis of a variety of compounds including terpenes and methyl ketones, whose diversity distinguishes not only species but also accessions within a species [8–10]. One particular feature of tomato type VI trichomes is the biosynthesis of terpenes from cisoid isoprenyl diphosphates. In *S. lycopersicum*, neryl diphosphate (NPP) is the substrate for monoterpenes such as α-phellandrene, and in *S. habrochaites* a range of sesquiterpenes such as α-santalene, bergamotene and zingiberene, are produced from *Z,Z*-FPP [11, 12]. There is substantial evidence that the metabolites produced by the various types of tomato glandular trichomes provide a primary defense barrier against a range of plant pests [13]. Wild tomato species in particular consistently display towards a number of arthropod pests an increased resistance which can be traced to the trichome secretions [14–18]. Interspecific crossing allows introducing trichome traits from the wild species into *S. lycopersicum*. The selection of these multigenic traits, however, requires in-depth knowledge not only of the genes commanding the biosynthesis of the metabolites, but also of the genes that determine trichome density, trichome architecture and the capacity to produce and secrete large amounts of the metabolites. There is indeed evidence that the quantity of metabolites produced influences the level of resistance [19]. While significant progress has been made on the elucidation of trichome specific metabolite pathways in the last years [2, 20], other aspects of glandular trichome biology remain largely unexplored at the molecular genetic level, such as the differentiation of the glandular cells or the transcriptional regulation of biosynthesis pathways. Furthermore, there is partial evidence that the regulatory mechanisms controlling glandular trichome development may not be the same as those for non-glandular trichomes [21], although initiation mechanisms are likely to be shared [22]. Therefore, one can anticipate that the extensive knowledge on Arabidopsis trichome development and differentiation may not be directly transferable to species with glandular trichomes.

To establish tomato as a model system for the study of glandular trichome development and differentiation, these processes should be first described precisely and categorized in specific stages. Using a variety of microscopy techniques we assemble here a detailed sequence of the development stages of type VI trichomes in the wild species *S. habrochaites* LA1777 and in *S. lycopersicum* LA4024. Our observations point to a highly reproducible and determined set of events leading to the formation of dedicated glandular structures with specific structural features, and provide a framework for further molecular studies of glandular trichome development and differentiation in tomato.

## Results

### The difference in external appearance of type VI glandular trichomes in *S. habrochaites* and *S. lycopersicum* is reflected by a distinct internal architecture

**Fig. 1:**
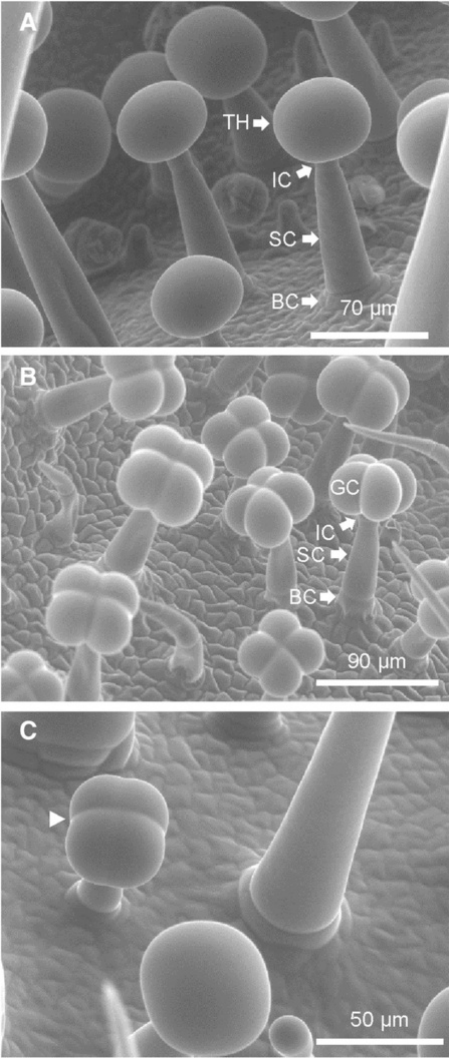
Scanning electron microscopy of type VI trichomes in *S. habrochaites* LA1777 (**a** and **c**) and *S. lycopersicum* LA 4024 (**b**). *BC* basal cell, *SC* stalk cell, *IC* intermediate cell, *TH* trichome head, *GC* glandular cell. The arrow head in (**c**) indicates the cell separation visible at an earlier development stage

**Fig. 2:**
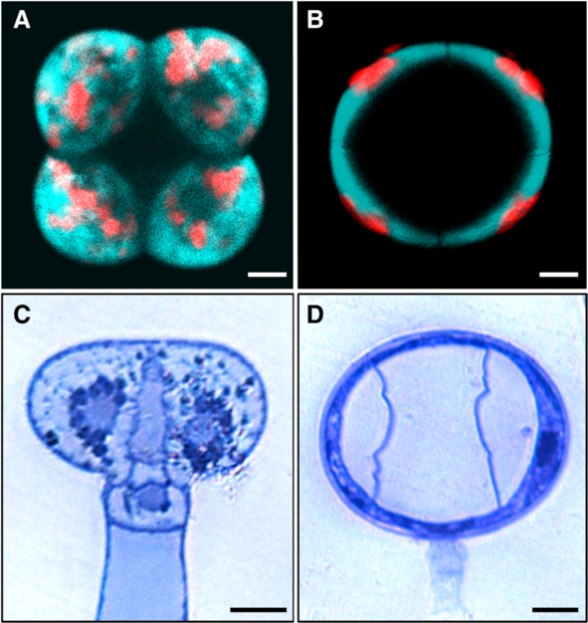
Fluorescence and bright field microscopy of type VI trichome heads from *S. habrochaites* and *S. lycopersicum*. **a**-**b** Fluorescence microscopy images of trichome heads of *S. lycopersicum* LA 4024 (**a**) and *S. habrochaites* LA 1777 (**b**). Bright field microscopy images of sections of type VI trichomes attached to the leaves and stained with toluidine blue from *S. lycopersicum* LA 4024 (**c**) and *S. habrochaites* LA 1777 (**d**). The *horizontal bars* correspond to 10 μm in all panels

There are a number of reports that document a higher metabolic productivity of glandular trichomes in the wild tomato species *S. habrochaites* compared to its cultivated relative *S. lycopersicum* [18, 23]. Two factors can contribute to this difference: a higher density of trichomes and a higher metabolic activity per trichome. We estimated the number of type VI glandular trichomes per leaflet (*n* = 5) on the adaxial side at 2573 ± 161 cm^−2^ in *S. habrochaites* versus 611 ± 171 cm^−2^ in *S. lycopersicum* as measured on leaflets that have an area of 1.6 ± 0.2 cm^2^ and 2.1 ± 0.9 cm^2^ respectively. However, this alone cannot account for the large difference in the content of metabolites produced by the trichomes which in absolute quantities can exceed 100 fold. Indeed, the amount sesquiterpene carboxylic acids produced by *S. habrochaites* LA1777 can reach up to 12 mg g^−1^ FW [19], whereas foliar concentrations of rutin, the most abundant secondary metabolite produced by glandular trichomes in *S. lycopersicum*, range from 70 to 170 μg.g^−1^ FW [24]. It was already observed that type VI trichomes in *S. habrochaites* and *S. lycopersicum* have a different appearance [25]. In *S. habrochaites* the glandular head looks round while in *S. lycopersicum* the contour of four glandular cells can be clearly distinguished. We confirm this difference in shape based on observations made with an environmental scanning electron microscope (ESEM) (Fig. 1). The type VI trichomes of both species have an identical overall architecture with a glandular head, an intermediate cell and a single stalk cell connecting the trichome to the leaf. The trichome sits on top of a single basal cell, whose diameter is slightly larger than that of the stalk cell (Fig. 1). Glandular cells in the round trichomes of *S. habrochaites* cannot be distinguished from the outside. But the presence of furrows at earlier stages of development (Fig. 1c) indicates that type VI trichomes of *S. habrochaites* are likely to contain four glandular cells as well. Thin sections of trichomes attached to leaves and direct observations of trichomes on the leaf surface with a fluorescence microscope confirm the presence of four glandular cells in both species (Fig. 2a and b). In addition, these images reveal the presence in *S. habrochaites* of a large intercellular space where metabolites can accumulate. In contrast, the type VI trichomes of the cultivated tomato have either no or a very small intercellular space, thus leaving little room for the storage of metabolites. ESEM pictures from shoot apex and developing leaves indicate the presence of fully mature trichomes already on leaf primordia (Additional file 1: Figure S1). Trichomes appear first on the abaxial side and subsequently on the adaxial side, where they end up being much denser (Additional file 1: Figure S1). This indicates that trichome initiation and differentiation occur at different times in different locations within a single leaf. Therefore even very young leaves will contain a trichome population of mixed development stages.

To gain further insight into the development and architecture of type VI trichomes, we carried out a number of experiments using fluorescence and electron microscopy, as well as immuno-stainings which will be presented in the following sections.

### Fluorescence microscopy of detached type VI trichomes

**Fig. 3:**
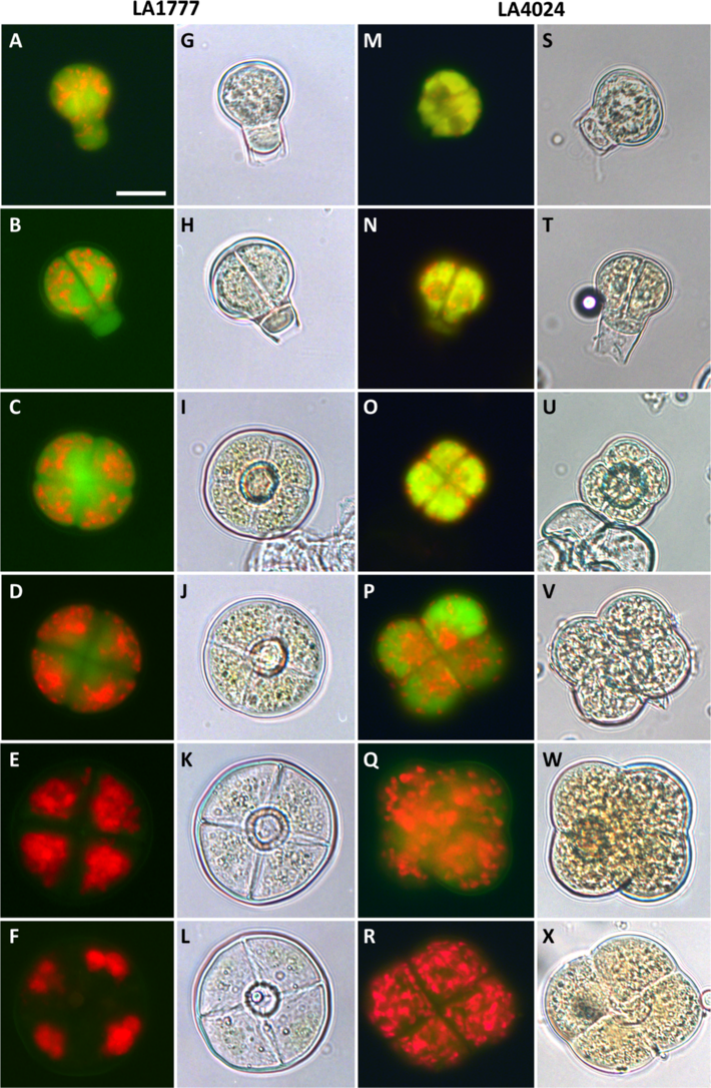
Fluorescence and bright field microscopy of detached type VI trichome heads from *S. habrochaites* LA 1777 and *S. lycopersicum* LA 4024. **a**–**f** Fluorescence microscopy (excitation 450–490 nm, emission 515 nm) of detached type VI trichomes from *S. habrochaites*. **g**–**l** Corresponding bright field microscopy of the fluorescence images shown in panels **a**-**f**. **m**-**r** Fluorescence microscopy of detached type VI trichome heads of *S. lycopersicum*. **s**–**x** Corresponding bright field microscopy of the fluorescence images shown in panels **m**–**r**. The horizontal bar in A represents 20 μm and applies for all images of the figure

Trichomes are difficult to observe in close range directly onto the leaf because of the irregular surface of the leaf and the tendency of trichomes to break easily. Thus, we first isolated them using a glass bead beating procedure (see Materials and Methods) inspired from previously published methods [26, 27]. We carried this out both for the cultivated tomato (LA4024) and the wild tomato (LA1777). The isolated trichomes were then directly observed with a fluorescence microscope without further treatment. Distinct development phases can be observed as illustrated in Fig. 3. In both species, the patchy red autofluorescence indicates the presence of chloroplasts throughout the development phases. Over 99 % of the trichomes are at the mature stage, i.e. with four clearly individualized glandular cells (Fig. 3f, l, r and x). This figure however is likely to be an overestimate because the heads of mature trichomes tend to break more easily than those of young trichomes. This is confirmed by the fact that the heads of young trichomes with only one or two pre-secretory cells are always seen with the intermediate cell (Fig. 3g, h, s and t) whereas this cell is not present in detached mature trichomes. In *S. lycopersicum*, the chloroplasts of trichomes in the mature stage seem equally distributed indicating that the cells occupy most of the volume of the trichome head (Fig. 3r). In contrast, in *S. habrochaites*, the plastids seem to aggregate at the periphery and are totally absent from the center, delineating the inter-cellular cavity between the glandular cells (Fig. 3f). The difference between the two species is most apparent in the later stages of development, where the constriction between the glandular cells is clearly marked in *S. lycopersicum* (Fig. 3p-r and v-x) whereas in *S. habrochaites* the four cells form a rounded shape (Fig. 3d-f and j-l). In the earlier stages, the development follows a very similar path in both species. With this preparation method the earliest stages we could isolate have one cell at the tip surmounting the intermediate cell (Fig. 3a, g, m and s). At that stage the developing trichome head has a diameter of approximately 20 μm. The single cell at the tip undergoes two successive divisions without significant enlargement, leading to a 4-cell head of around 25–30 μm diameter (Fig. 3c, i, o and u). The trichome head then significantly enlarges to reach a size of around 60 μM. The average diameter is 69.5 μm ± 6 (*n* = 50) in *S. habrochaites* and 57.0 μm ± 4.9 (*n* = 50) in *S. lycopersicum*. In exceptional cases (less than 0.5 %) trichome heads of over 100 μm could be detected in *S. habrochaites* (Additional file 1: Figure S2). Observations with a fluorescence microscope of live trichomes on the leaf surface reveal that in the most mature stages, the inter-cellular cavity of the trichome head in *S. habrochaites* occupies around 65 % of the total volume (Fig. 2b), with the remaining cytoplasm forming a thin layer at the periphery of the trichome head. Since in *S. habrochaites*, the head cells seem to enlarge before the inter-cellular cavity is formed, this indicates that the intracellular volume dramatically decreases as the trichome matures.

### Transient accumulation of a flavonoid-derived fluorescent substance in the early stages of trichome development

**Fig. 4:**
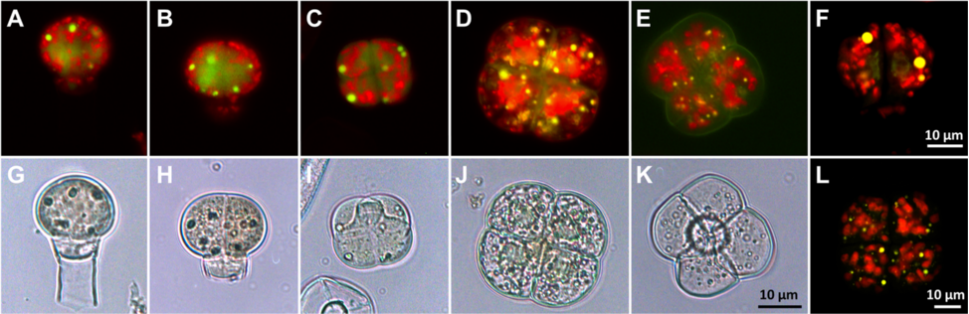
Fluorescence and bright field microscopy of detached type VI trichome heads from *S. lycopersicum* LA 1049 carrying a mutation in a chalcone isomerase gene. **a**–**e** Fluorescence microscopy (excitation 450–490 nm, emission 515 nm) of detached type VI trichomes from *S. lycopersicum* LA1049. **g**–**k** Corresponding bright field microscopy of the fluorescence images shown in panels **a**–**e**. **f** and **l** laser scanning microscopy images of a 2-cell stage (**f**) and a 4-cell stage (**l**) trichome head of LA1049

One striking feature common to both species is the presence of an intense yellow-green auto-fluorescence in the early stages of development, which progressively decreases after the second division to completely disappear in the mature stages (Fig. 3 and Additional file 1: Figure S3). The fluorescence spectrum is reminiscent of a flavonoid-type compound (Additional file 1: Figure S4), although it should be noted that flavonoids typically are poorly auto-fluorescent. Thus it is unlikely that this unknown substance is an unmodified flavonoid. Due to the highly transient presence of the compound and the small numbers of trichomes at early stages of development we have not been able to isolate sufficient quantity of material to identify the compounds yet. Interestingly, in a recent manuscript, tomato mutants in a gene encoding chalcone isomerase (CHI) have smaller type VI trichomes which produce significantly less monoterpenes [28]. Since CHI is a key enzyme in the biosynthesis of flavonoids, it was of particular interest to determine if this fluorescence observed in the WT is still present in this mutant (*Solanum lycopersicum* accession LA1049). Therefore, trichomes from LA1049 plants were isolated and observed with a fluorescence microscope. Remarkably, the yellow fluorescence is now concentrated in discrete spots (Fig. 4), which are also visible in light microscopy suggesting the fluorescence is concentrated in vesicle-like structures. Observations with a confocal microscope indicate that these vesicles are localized within the cells (Fig. 4f and l). Furthermore, this fluorescent material is visible throughout the development of the trichomes including in trichomes with four head cells, whereas in the wild type, the fluorescence disappears in the mature stages. These observations confirm that the fluorescent compound seen in the wild type is a flavonoid and that it plays a critical role in the correct differentiation of the type VI glandular trichomes into active terpene-secreting structures.

### The junction between the head cells and the intermediate cell constitute a fragile point facilitating the release of metabolites

**Fig. 5:**
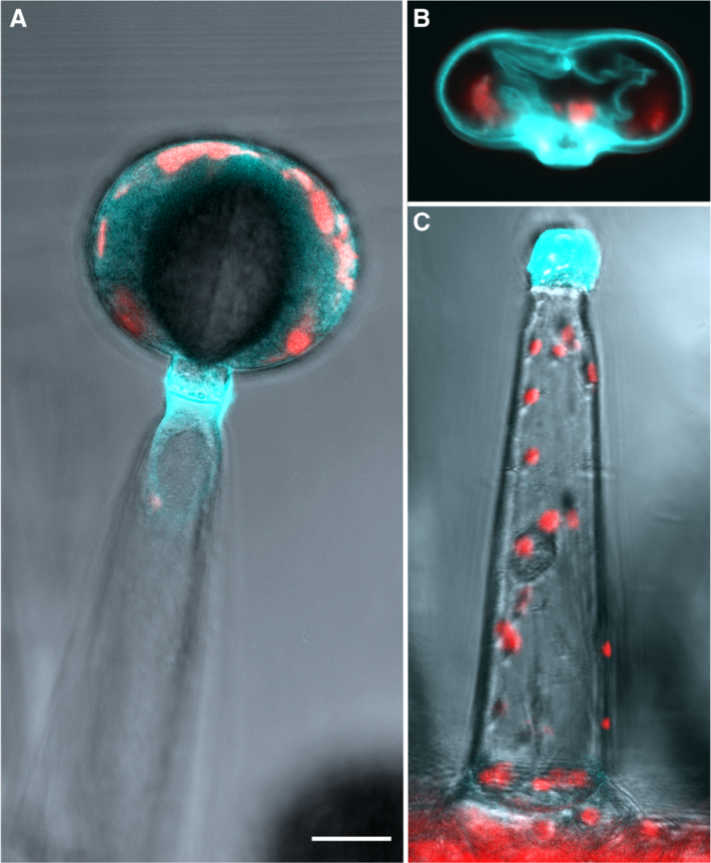
Fluorescence microscopy of live *S. habrochaites* type VI trichomes. **a** Whole type VI trichome. **b** Detached type VI trichome head. **c** Stalk and intermediate cells of a decapitated type VI trichome. The horizontal white bar represents 20 μm

We noted that when type VI trichomes are collected, only the head cells are present, except in the earliest development stages, when the head contains only one or two cells, which remain attached to the intermediate cell. In *S. habrochaites*, trichomes that are still bound to the leaf have a perfectly round appearance (Fig. 5a). In contrast, detached trichome heads appear as though they are collapsed, with the cell walls between the glandular cells having a wavy appearance (Fig. 5b). These observations point to the role of the intermediate cell as a plug preventing the release of metabolites form the inter-cellular cavity. Furthermore, this also indicates that the junction between the intermediate cell and the head cells constitutes a fragile point favoring the release of the metabolites when the trichomes are physically damaged, for example by an herbivore. Confirmation of this hypothesis was provided by the observation of decapitated trichomes by fluorescence microscopy showing that the breakage takes place between the four head cells and the intermediate cell (Fig. 5c). The intermediate cell exhibits a strong blue fluorescence indicative of the presence of phenolic compounds in the cell wall, and remains attached to the stalk cell. Further evidence of the junction between the head cells and the intermediate cell as a fragile point is provided by electron microscopy images (see below).

### Ultrastructure of type VI trichomes

To delve deeper into the structure of the type VI trichomes, ultra-thin sections of apices were observed by transmission electron microscopy. Images of the trichomes from LA4024 and LA1777 still attached to the leaves and at different development stages could be observed and are described below.

**Fig. 6:**
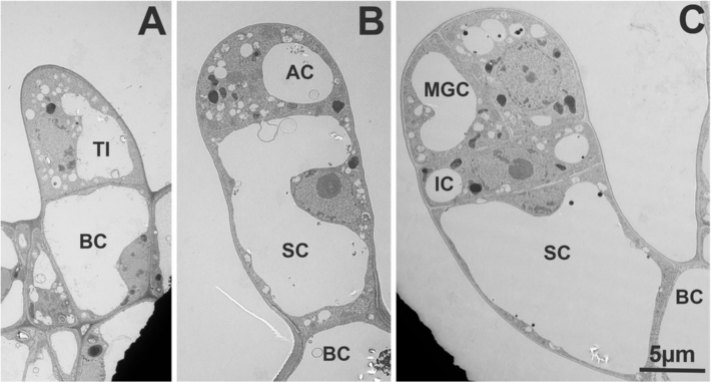
Electron microscopy of early stages of type VI glandular trichome development in *S. habrochaites*. **a** Trichome initial with a basal cell (BC) and the initial cell (TI). At this stage it is not possible to distinguish the various trichome types. **b** Trichome initial with one apical cell (AC) and a stalk cell (SC). **c** young trichome with an intermediate cell (IC) and two mothers of glandular cells (MGC)

**Fig. 7:**
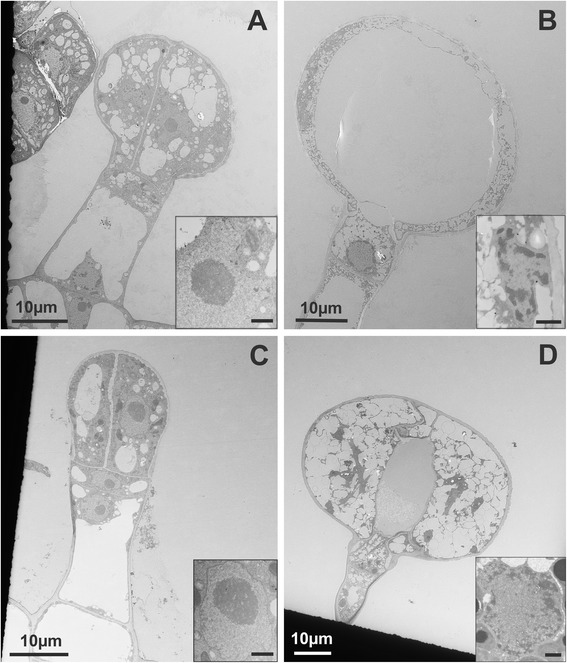
Electron microscopy of type VI trichomes at different stages of development. **a**-**b** *S. habrochaites* LA 1777 trichomes. **a** a young 4-cell stage trichome. **b** mature trichome. **c**-**d** *S. lycopersicum* LA 4024 trichomes. **c**: a young 2-glandular cell trichome. **d** Mature trichome. Inserts in **a**-**d**: ultrastructure of nuclei of glandular cells in the corresponding stage of development. The scale bar in the inserts represents 1 μM

The earliest stages of trichome initiation can be traced back to enlarged epidermal cells which bulge out of the surface (Fig. 6a). At this stage, it is not really possible to distinguish between the different types of trichomes that will emerge from these trichome initials. The first signs pointing to the development of type VI trichomes is a first unequal division of the trichome initial, resulting in a small apical cell and the future stalk cell (Fig. 6b). The apical cell divides once again unequally, giving rise to an apical cell and the intermediate cell, although this stage could not be directly observed. As seen in the fluorescence microscopy, the apical cell then undergoes a first round of anticlinal division (Fig. 6c) followed by another giving rise to the four glandular cells. This pattern is identical in *S. lycopersicum* and in *S. habrochaites*, indicating a similar developmental program in both species. In the early stages, the apical and the intermediate cells have a dense cytoplasm with one larger vacuole and a few small vacuoles. In contrast the stalk cell has a large vacuole which occupies the majority of the cell volume. All major organelles can be clearly distinguished in the early stages in the apical and intermediate cells, including mitochondria and plastids, where starch granules can also be observed (Fig. 7a, c and Additional file 1: Figure S5). At these stages the nuclei are large and display a central nucleolus in all cells (inserts Fig. 7a, c).

**Fig. 8:**
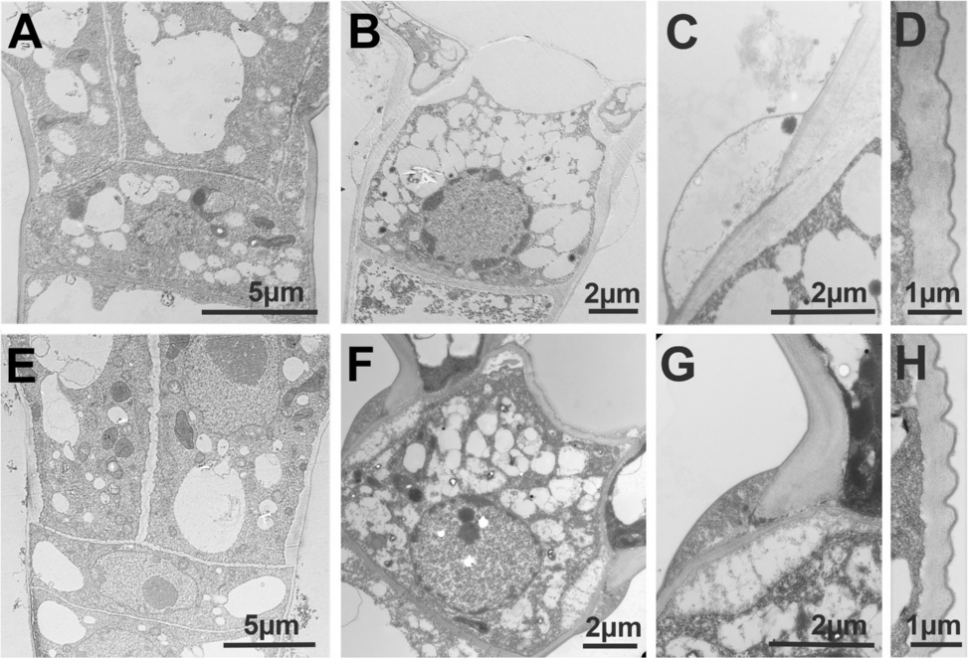
Electron microscopy of type VI trichomes of *S. habrochaites* and *S. lycopersicum*. **a**–**d** *S. habrochaites*. **e**–**h** *S. lycopersicum*. Intermediate cell at an early 4-cell stage (**a**) or 2-cell stage (**e**) and at the mature 4-cell stage (**b**, **f**). **c** and **g** Junction between the intermediate cell and the glandular cells highlighting the deposit of extra-cellular material and the breaking point. **d** and **g** External cell wall and cuticle of the glandular cells

Mature trichomes have common and distinguishing features between the two species. In both species, the number of small vacuoles is increasing and progressively filling up most of the cellular space in the glandular and in the intermediate cells (Fig. 7b and d), although there are more and smaller vacuoles in *S. habrochaites* than in *S. lycopersicum*. In the intermediate cell the nucleus is still clearly visible, but the nucleolus is much smaller. Instead, dark nuclear material is now accumulating at the periphery lining the nuclear envelope, which typically corresponds to clumps of condensed chromatin (Fig. 8b and f). These changes seem even more pronounced in the nuclei of the secretory cells of mature trichomes (inserts of Fig. 7b and d). The amount of condensed chromatin in the mature trichomes seems to be much larger than in young trichomes, indicating extensive remodeling of chromosome architecture during trichome differentiation and maturation. Another common feature is the presence of a lump of extracellular material exactly at the junction between the intermediate and the glandular cells (Fig. 8b-c and f-g). This material covers the thick cell wall of the glandular cells and the thinner wall of the intermediate cell. The boundary between the two cell walls is also clearly visible, likely delineating the position where the separation occurs (Fig. 8c and g). Although this material has a different appearance between *S. lycopersicum* and *S. habrochaites*, its position and distribution across the boundary between the glandular and the intermediate cells suggest a similar role. The accumulation of such extra cell wall material can be seen in abscission zones [29]. In both species, the chloroplasts in the mature trichomes do not have recognizable thylakoid membranes (Additional file 1: Figure S5B and F). Instead, they contain darker staining patches, particularly in LA4024, which have no apparent organized structure and seem to be squeezed between the dense network of small vacuoles or vesicles. Whether the chloroplasts are degenerating or have an organization which is specific to the trichomes remains to be determined. One major difference between the two species is the size of the inter-cellular space. In *S. habrochaites*, this space is now occupying a large part of the volume of the glandular head (estimated at 65 %). There is hardly any electron absorbing material, indicating that during preparation of the material for the sections the metabolites that are stored therein have been released or that the metabolites are electron-transparent, but ruling out the presence of polymeric material that would be bound to the cell wall. This cavity is likely to have been formed through hydrolysis of the internal cell wall separating the glandular cells, and remains of this cell wall can still be seen as debris in the earlier stage (Additional file 1: Figure S5C and D) and as a wavy thread going from the top to the bottom of the space in the mature stages (Fig. 2d and Additional file 1: Figure S5D). In contrast, in *S. lycopersicum* the glandular cells still occupy the majority of the volume, although a small intercellular space is clearly visible. Here, electron absorbing material can be seen, suggesting that cell wall hydrolysis is not as extensive as in *S. habrochaites* (Fig. 7d). This is also supported by the fact that the internal cell walls lining this space are significantly thicker than in *S. habrochaites* (Fig. 7d).

Observation of the intermediate cell also reveals common and distinct features between the two species. Plasmodesmata at the proximal side, i.e. between the intermediate and the stalk cells, can be seen in both species (Fig. 8b and f, for details see Additional file 1: Figure S5G). The distal side of the intermediate on the other hand shows differences. In *S. habrochaites*, the area in contact with the cavity and interfacing the glandular cells appears like an empty space likely to be extra-cellular (Fig. 8b). This space is delimited by a membrane which in a few places comes into contact with the glandular cell. The cell wall lining this area appears loose and stains poorly, indicating a possible degradation. In the corresponding area in *S. lycopersicum*, a well-structured cell wall is visible, with the presence of plasmodesmata, although not as clearly visible as in the proximal side (Additional file 1: Figure S5H). This could be due to the orientation of the section however. These observations point to common mechanisms for the communication between the intermediate and the stalk cell but to different characteristics concerning the communication between the intermediate and the glandular cells.

Another feature of interest is the outer envelope lining the glandular cells. In both species it has a darker staining thin edge, possibly corresponding to the waxy layer of the cuticle and a more lightly stained thick internal part likely representing cell wall material. In the early stages, it is already quite thick (between 0.6 and 0.8 μM) (Fig. 7a and c) although the cells will significantly enlarge. In mature cells, it has the same thickness, indicating there must have been deposition of material to adjust to the increased surface to cover (Fig. 8d and h).

### Immuno-labelling of cell wall components

**Fig. 9:**
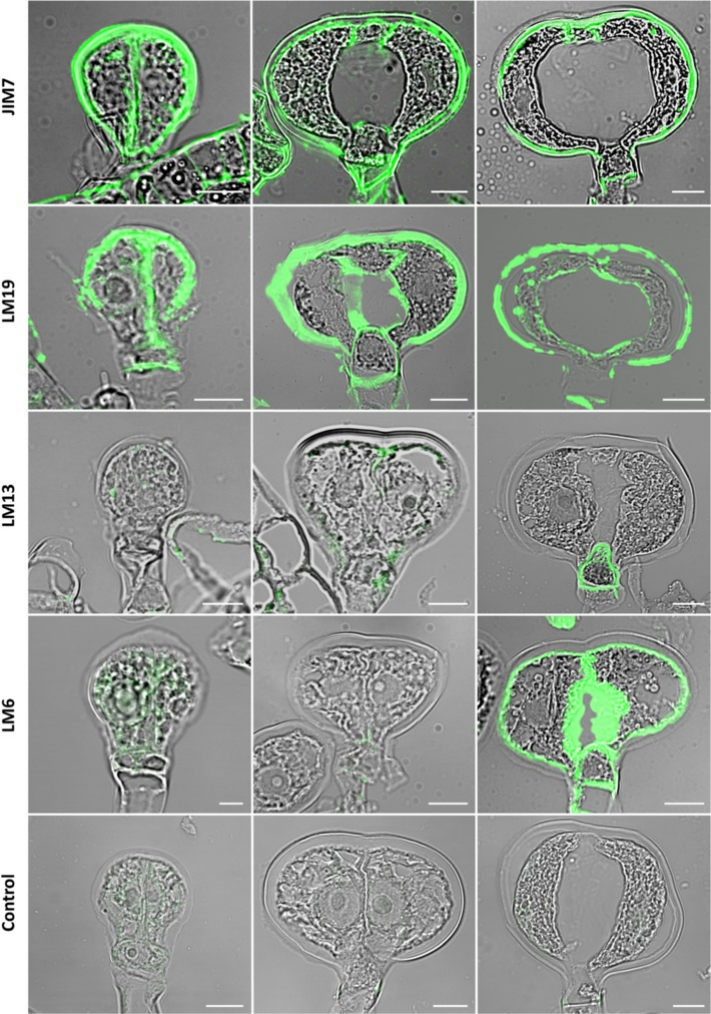
Immuno-labelling of cell wall components of type VI trichomes from *S. habrochaites* LA1777. The antibodies used are indicated on the left (for details see text). *Left panels*: 1 to 2-cell stage trichomes. Middle panels: immature 4-cell stage trichomes. *Right panels*: mature 4-cell stage trichomes. The control is from labelling done with the secondary antibody alone. The white horizontal bar represents 10 μm

The presence of an intercellular cavity and of a thick envelope indicates that specific cell wall metabolism and remodeling events are likely to take place during the development of type VI trichomes of *S. habrochaites* LA1777. To get a first insight into these aspects, several commercially available antibodies recognizing distinct cell wall components were used for immuno-labeling followed by fluorescence microscopy (Fig. 8). The sections were carried out on young leaves in order to observe the trichomes at different stages of development and still attached to the leaves. Methyl-esters of pectin are recognized selectively by JIM7 while it does not bind un-esterified homogalacturonan. JIM7 labelling consistently results in strong labelling of the outer cell wall of the developing and mature trichomes, while the inner cell wall only gives a light signal during the very early stages of development (Fig. 9, top row). In contrast, labelling with LM19, which preferentially recognizes un-esterified homogalacturonan but also binds esterified pectin, gives a strong signal in the inner cell wall, particularly at stages during which formation of the cavity is emerging (Fig. 9, second row from the top and Additional file 1: Figure S6). Thus, pectin demethylation seems to play a role for the lysis of the inner cell wall and formation of the inter-cellular cavity. LM13 specifically recognizes arabinan polymers and gives a striking pattern with a strong labelling of the intermediate cell specifically in the mature stage (Fig. 9, third row from the top and Additional file 1: Figure S6). Finally, LM6 which in addition to arabinans also recognizes arabinogalactan proteins (AGPs) gives a signal on the outer cell wall and a very strong signal in the inner cell wall also at the mature 4-cell stage (Fig. 9, fourth row). This indicates that AGPs are constituents of the outer and particularly the inner cell walls.

## Discussion

### Type VI glandular trichomes have a highly reproducible architecture

Our observations based on ESEM, fluorescence microscopy and sections analyzed by light microscopy or transmission electron microscopy point to an invariable architecture of the type VI trichomes in both species. It consists of a stalk cell, an intermediate cell and the four glandular cells all connected to the intermediate cell. The glandular cells emerge via a succession of two equal anticlinal divisions, leading to the characteristic disposition of the glandular cells on one plane. This overall architecture is strongly reminiscent of that of the peltate trichomes of peppermint, which is representative of the Lamiaceae [30]. The stalk cell of Turner et al. [30] corresponds to our intermediate cell and our stalk cell to their basal cell. We proposed this nomenclature because the intermediate cell in type VI trichomes does not elongate, whereas the stalk cell significantly elongates to bring the glandular cells to an elevation of 200–300 μm above the epidermis. In contrast, the peppermint trichomes are lodged into small depressions of the epidermis, so that the peltate trichomes are almost level with the epidermal surface. Furthermore peppermint trichomes typically have 8 glandular cells, indicating an additional round of division. However, sweet basil (*Ocimum basilicum*) peltate trichomes have four glandular cells [31], indicating there is species-to-species variation in the number of cell divisions. Another striking difference is the absence of a subcuticular storage space in the tomato trichomes, a feature which is characteristic of the peltate trichomes of the Lamiaceae [32]. Instead, the type VI tomato trichomes have an intercellular cavity of varying size depending on the species (see below). Despite these differences, there are many common features between the tomato and Lamiaceae trichomes, and the invariable architecture of these organs makes it a particularly valuable material for research in plant developmental biology. The tomato genus, with its excellent genetics and recently sequenced genomes, represents therefore an appropriate model system for detailed investigation of the glandular trichome developmental genetics.

### The early stages of trichome differentiation are marked by the transient presence of an auto-fluorescent flavonoid

Strikingly this fluorescence disappears as the trichome enters the final stage of its differentiation, namely when the four glandular cells enlarge to reach their final size. Although the identity of the fluorescent compounds could not be determined, the fluorescence spectrum is reminiscent of a flavonoid-like substance (see Additional file 1: Figs. S3 and S4). Interestingly, a recent publication shows that a tomato chalcone isomerase (CHI) mutant (*S. lycopersicum* accession LA1049) has smaller type VI trichomes accompanied by a largely reduced amount of terpenes produced in the trichomes [28], which in turn leads to hypersensitivity to insect herbivores. Kang et al. proposed several hypotheses to account for this intriguing connection between flavonoid and terpenoid metabolism, one of them being that of a metabolic feedback control due to the fact that these two pathways have in common phosphoenolpyruvate as a precursor. We found that in this mutant the fluorescence has a completely different distribution and accumulates in discrete vesicles (Fig. 4). Images obtained with a confocal microscope indicate that these vesicles are located within the cells (Fig. 4f and l). These observations strongly supports the flavonoid nature of the fluorescent compound seen in the WT. Also, the fluorescence is no longer transient as in the WT, but remains even in the mature trichomes of LA1049 (Fig. 4e and l). The transient presence of this compound in the WT does not really support the metabolic feedback control proposed by Kang et al. [28], since it tends to disappear when the trichomes are in the active secretory phase (4 cell stage). Its transient presence would rather indicate a function in the late differentiation of glandular trichomes rather than a direct metabolic connection with the terpenoid pathway.

Flavonoid normally fluoresce poorly, but there are a few reports of strongly auto-fluorescent compounds of this class. All these reports point to a modification in the 5 position. 5-*O*-methyl quercetin derivatives such as azalein or azaleatin were isolated from *Plantago* and *Rhododendron* species [33, 34], and 5-*O*-glucosides of various flavonoids could also be identified in various plant species [35]. Interestingly, cocoons of the silkworm, *Bombyx mori*, are able to glycosylate quercetin at the 5 position, and the resulting fluorescent compounds were shown to provide reinforced UV-protection of the cocoons [36]. Further work will be needed to see if the fluorescent substance present in tomato trichomes is indeed a 5-*O*-glycosylated or –methylated flavonoid.

### Type VI glandular trichomes have an intercellular storage cavity which is significantly enlarged in *S. habrochaites*

Thin sections and observations under a fluorescence microscope of type VI glandular trichomes revealed the presence of an intercellular space between the 4 glandular cells. Interestingly, this cavity is greatly enlarged in *S. habrochaites*. In mature trichomes of *S. habrochaites*, we estimate that up to 65 % of the trichome head consists of this intercellular cavity, which represents a significant storage space for metabolites that are produced in the glandular cells. This difference between the two species also explains why *S. habrochaites* accumulates significantly more secondary metabolites (essentially sesquiterpene carboxylic acids) than *S. lycopersicum*, and thereby contributes to the increased resistance against herbivores of this species. This cavity emerges after the second cell division of the terminal cell, i.e. when the four glandular cells are present. This type of intercellular cavity in secretory trichomes is rare but similar cases have already been described previously for example in various species of *Rhododendron* [37]. Its genesis is highly controlled and requires degradation of the internal cell wall common to the glandular cells but only over the central and bottom part. The four glandular cells are still attached to each other at the top, whereas at the bottom the intermediate cell ensures the cohesion and constitutes a plug to prevent the release of the metabolites. As shown by immuno-labelling, this selective cell wall degradation is associated with the presence of de-methylated pectin. The methylation status of pectin plays a critical role in the remodeling of plant cell walls. The separation of the microspore tetrads for example, requires the demethylation of pectin as demonstrated in a pectin-methyl esterase (PME) mutant leading to the formation of the typical *quartet* phenotype in *Arabidopsis thaliana* [38].

### The junction between the glandular cells and the intermediate cell constitutes a micro-abscission zone: an adaptation for the rapid release of metabolites

We noted that the mature trichomes invariably break at the same position when they are harvested: the intermediate cell stays attached to the stalk cell while the four glandular cells stick together. In *S. habrochaites*, this breakage leads to a collapse of the trichome head as can be seen from the wavy appearance of the cell walls and the flattened cells. This indicates that the metabolites contained in the inter-cellular cavity exert a pressure which is released when the head breaks off. A light touch is sufficient to trigger trichome decapitation. This can be seen as an adaptation for the sudden release of metabolites unto herbivores which come in contact with the trichomes. Structural features underlie this adaptation. First, a ring of secretory material can be seen at the outer junction between the intermediate and the glandular cells. In Arabidopsis lines overexpressing the floral abscission regulating gene *IDA* (*INFLORESCENCE DEFICIENT IN ABSCISSION*), large amounts of arabinogalactan protein (AGP) are deposited around abscission zones [29]. Interestingly, immuno-labelling with LM13, a monoclonal antibody specifically recognizing arabinans, gives a strong signal restricted to the intermediate cell. Whether the material accumulating around the breakage point of the trichome is made of arabinogalactan protein remains to be shown however. Second, a distinct boundary can be seen between the thick cell wall of the glandular cells and the thinner cell wall of the intermediate cell. It is possible that the outer material holds the two cell types together and breaks up upon a light touch.

## Conclusions

The development of type VI glandular trichomes in the two tomato species share a number of common features, mostly during the early stages. Differences start being clearly visible when the four glandular cells are in place. *S. habrochaites* differs essentially with the formation of the large intercellular cavity, the smooth round appearance of the trichome head and the localization of the chloroplasts at the periphery of the glandular cells. Our observations point to a highly regulated development and differentiation programme that includes a strict cell division sequence, polarized and localized cell wall lysis and remodeling, and the formation of a micro-abscission zone. These results establish a foundation for future studies, which will consist in performing stage specific transcriptome and metabolome analyses.

## Methods

### Plant material and growth conditions

Seeds of accession LA1777 (*S. habrochaites*) and LA4024 (*S. lycopersicum*) are from the Tomato Genetics Resource Center, UC Davis, USA. Plants were grown in a greenhouse with controlled conditions of 25 °C and 55 % humidity during the day, and 20 °C and 75 % humidity during the night. Plants were illuminated for 16 h (from 6:00 am to 10:00 pm) resulting in a light intensity of 5 to 25 Klux depending on whether conditions. Once a week, the plants were watered with a fertilizer solution (0.1 % Kamasol Brilliant Blau, Compo Expert GmbH, Germany). Leaf material for trichome harvests or microscopy observations was from apexes (including young leaves up to 1–2 cm) from plants aged 2 to 4 months.

### Harvest of glandular trichomes

Glandular heads from type VI trichomes were collected using a procedure modified from Schilmiller et al. [39]. Apices with a size of 1–2 cm from 2 to 4 months old plants were collected into a 250 ml glass bottle containing cold isolation buffer (200 mM sorbitol, 50 mM Tris-HCl, 20 mM sucrose, 10 mM KCl, 5 mM MgCl_2_, 5 mM succinic acid, 1 mM EGTA and 0.5 mM K_2_HPO_4_,) and 15 g of 0.75 – 1 mm glass beads. Type VI glands were isolated by shaking the bottle by hand for 2 min. Afterwards the material was filtered through two steel sieves of 150 and 63 μm meshes respectively to remove apices and glass beads. Finally the heads of the type VI trichomes were collected onto a 25 μm mesh steal sieve washed with the isolation buffer and concentrated by a short centrifugation.

### Environmental Scanning Electron Microscopy (ESEM)

Scanning electron micrographs of fresh, unfixed plant material were made with an ESEM XL-30 FEG (FEI/Philips, Eindhoven, The Netherlands) operating in the wet mode. The gas pressure in the ESEM chamber (1.5 mBar) was regulated by introducing water vapor. A gaseous secondary electron detector was used for imaging.

### Embedding of plant material in PEG 1500

Plant material for thin-sectioning was embedded using a method modified from Hause et al. [40]. Apices with a size of 0.5 cm of 1 month old plants were collected in 4 % para-formaldehyde/0.1 % Triton X-100 in PBS. Subsequently the plant material was vacuum infiltrated for 3 times for 5 min. Fixation was done for 2 h at room temperature under gentle shaking. The apices were then washed twice for 15 min in PBS and dehydrated at room temperature in a series of rising ethanol concentrations as follows: 30 min 10 % EtOH, 60 min 30 % EtOH, 60 min 50 % EtOH, overnight 70 % EtOH, 30 min 90 % EtOH, 30 min 100 % EtOH. The subsequent infiltration of PEG 1500 was done at 50 °C as follows, 30 min 100 % EtOH, 60 min 25 % PEG in EtOH, 60 min 50 % PEG in EtOH, 60 min 75 % PEG in EtOH, 60 min 100 % PEG in EtOH. The 100 % PEG solution was exchanged once and incubated for another 60 min before the embedding of the apices and the hardening of the embedded material at room temperature overnight.

### Light and fluorescence microscopy

Microscopy analysis of the developing trichomes was performed on isolated type VI heads. After isolation using the glass bead procedure described above, the trichomes were directly placed on a glass slide and observed in the brightfield- and in the fluorescence-mode (Filterset 9; excitation 450–490 nm; emission 515 nm LP) with an AxioImager Z1 microscope (Zeiss, Jena, Germany). Trichomes, on the leaves as well as isolated, were also observed with a LSM 710 microscope (Zeiss, Jena, Germany). Autofluorescence was excited at 405 nm and recorded at 420–545 nm (cell wall) and 645–735 nm (chloroplasts).

### Transmission electron microscopy

Leaves were fixed with 3 % glutaraldehyde (Sigma Aldrich, Taufkirchen, Germany) in sodium cacodylate buffer (SCB) pH 7.2 for 4 h at room temperature, washed with SCB, postfixed with 1 % osmiumtetroxide (Carl Roth, Karlsruhe, Germany) in SCB, dehydrated in a graded ethanol series, and embedded in epoxy resin [41]. After polymerization the material was sectioned with an Ultramicrotome S (Leica; Wetzlar, Germany). Ultrathin sections (80 nm) were transferred to formvar-coated grids and poststained with uranyl acetate and lead citrate. The sections were observed with a Zeiss Libra 120 transmission electron microscope operating at 120 kV (Carl Zeiss Microscopy, Oberkochen, Germany). Images were taken applying a Dual-Speed on axis SSCCD camera (BM-2k-120; TRS, Moorenweis, Germany).

### Immunostainings

Immunostainings were performed on PEG 1500 embedded material after a modified protocol of Verhertbruggen et al. [42]. Cross sections of 3 μm thickness were transferred to poly-Lysine (Sigma-Aldrich) coated glass slides and washed twice with PBS. After incubation with 0.1 M NH_4_Cl the glass slides were washed again and blocked with 5 % BSA in PBS for 30 min. Primary antibodies (LM6, LM13, LM19 and JIM7) are all rat monoclonal antibodies from PlantProbes (http://www.personal.leeds.ac.uk/~bmbjpk//pp/). They were added in 1:10 dilution and incubated at 4 °C overnight. After washing three times with 0.1 % BSA in PBS and one time with 1 % BSA in PBS glass slides were incubated with FITC-coupled anti-rat secondary antibody produced in goat (Sigma-Aldrich, catalog number F6258) at 1:100 dilution for 2 h at room temperature. Afterwards glass slides were washed four more times with PBS, dried and covered with 60 μl anti-fade solution (Citifluor). The stained sections were observed with a LSM 710 microscope (Zeiss, Jena, Germany) using the 405 nm laser line for excitation and recording emission at 505–570 nm.

## Additional file

Additional file 1: Figure S1. ESEM view of an apex from a *S. habrochaites* LA 1777 plant. **Figure S2.** Exceptionally large type VI trichomes in S*. habrochaites.* **Figure S3.** Fluorescence emission spectra of developing type VI trichomes in *S. habrochaites*. **Figure S4.** Comparison of the emission spectrum of developing type VI trichomes in *S. habrochaites* with that of known flavonoid substances. **Figure S5.** Transmission electron microscopy of type VI trichomes in *S. habrochaites* LA1777 (A-D) and *S. lycopersicum* LA4024 (E-F). **Figure S5.** Immuno-labelling of type VI trichomes from *S. habrochaites* LA1777. (PDF 1303 kb)

## Abbreviations

AGP: arabinogalactan protein
BSA: bovine serum albumine
CHI: chalcone isomerase
ESEM: environmental scanning electron microscopy
EtOH: ethanol
FITC: fluorescein isothiocyanate
FW: fresh weight
LSM: laser scanning microscope
NPP: neryl diphosphate
PBS: phosphate buffer saline
PEG: polyethylene glycol
PME: pectin methyl esterase
SCB: sodium cacodylate buffer
*Z,Z*-FPP: *cis,cis*-farnesyl diphosphate
