# Supplementary material for: The development of type VI glandular trichomes in the cultivated tomato Solanum lycopersicum and a related wild species S. habrochaites
Source: BMC Plant Biol. 2015 Dec 12;15:289. doi: 10.1186/s12870-015-0678-z (PMC4676884; doi:10.1186/s12870-015-0678-z)
Supplement: Additional file 1: Figure S1. — ESEM view of an apex from a S. habrochaites LA 1777 plant. Figure S2. Exceptionally large type VI trichomes in S. habrochaites. Figure S3. Fluorescence emission spectra of developing type VI trichomes in S. habrochaites. Figure S4. Comparison of the emission spectrum of developing type VI trichomes in S. habrochaites with that of known flavonoid substances. Figure S5. Transmission electron microscopy of type VI trichomes in S. habrochaites LA1777 (A-D) and S. lycopersicum LA4024 (E-F). Figure S5. Immuno-labelling of type VI trichomes from S. habrochaites LA1777. (PDF 1303 kb) [file 12870_2015_678_MOESM1_ESM.pdf]

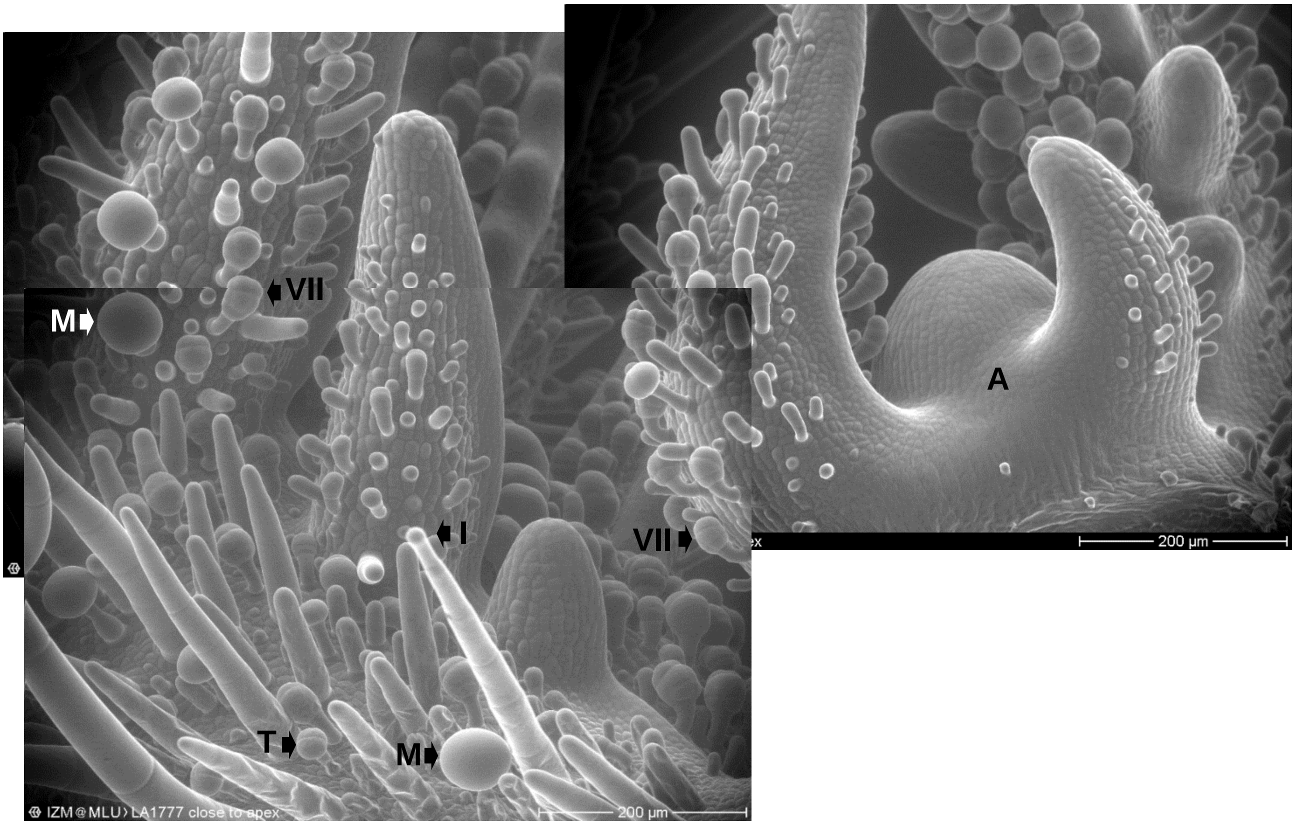

Supplemental Figure 1. ESEM view of an apex from a *S. habrochaites* LA 1777 plant. Three overlapping images were assembled. Type VI trichomes of different development stages can be observed. M: mature type VI trichome. T: two-glandular cell stage. Developing type I (I) and type VII (VII) trichomes can also be observed. A: Apex.

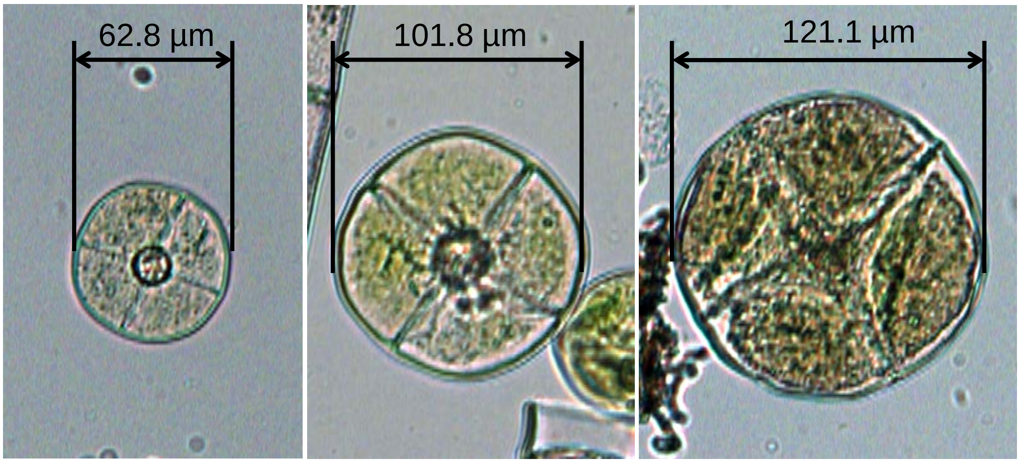

Supplemental Figure 2. Exceptionally large type VI trichomes in *S. habrochaites*.
The type VI trichome on the left panel is representative of the average size. Trichomes in the middle and right panels are exceptionally large trichomes.

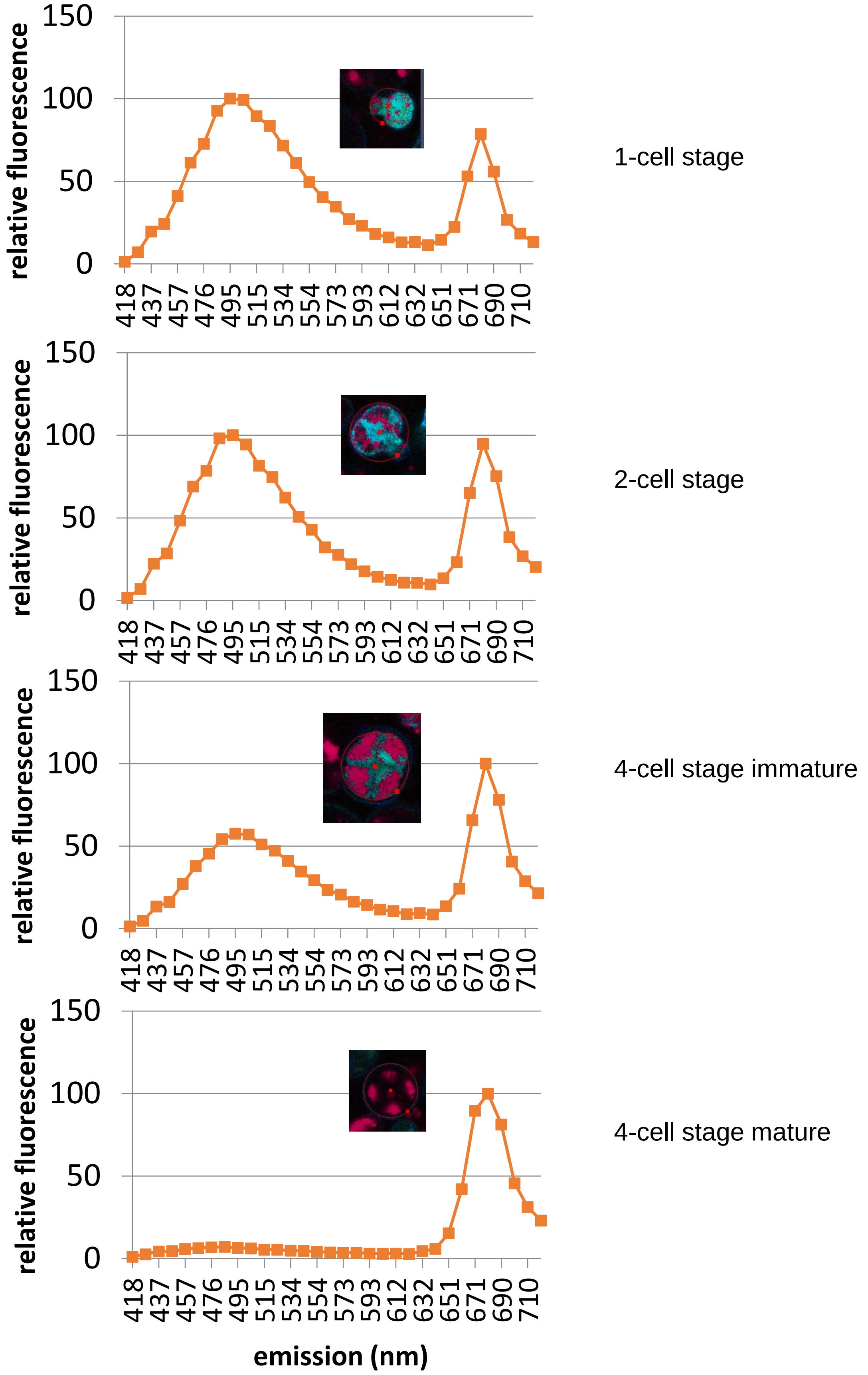

Supplemental Figure 3: Fluorescence emission spectra of developing type VI trichomes in *S. habrochaites*. The emission maximum at around 500 nm (white arrow) is highest in the early stages, goes down from the young 4-cell stage onwards and is absent from mature trichomes.

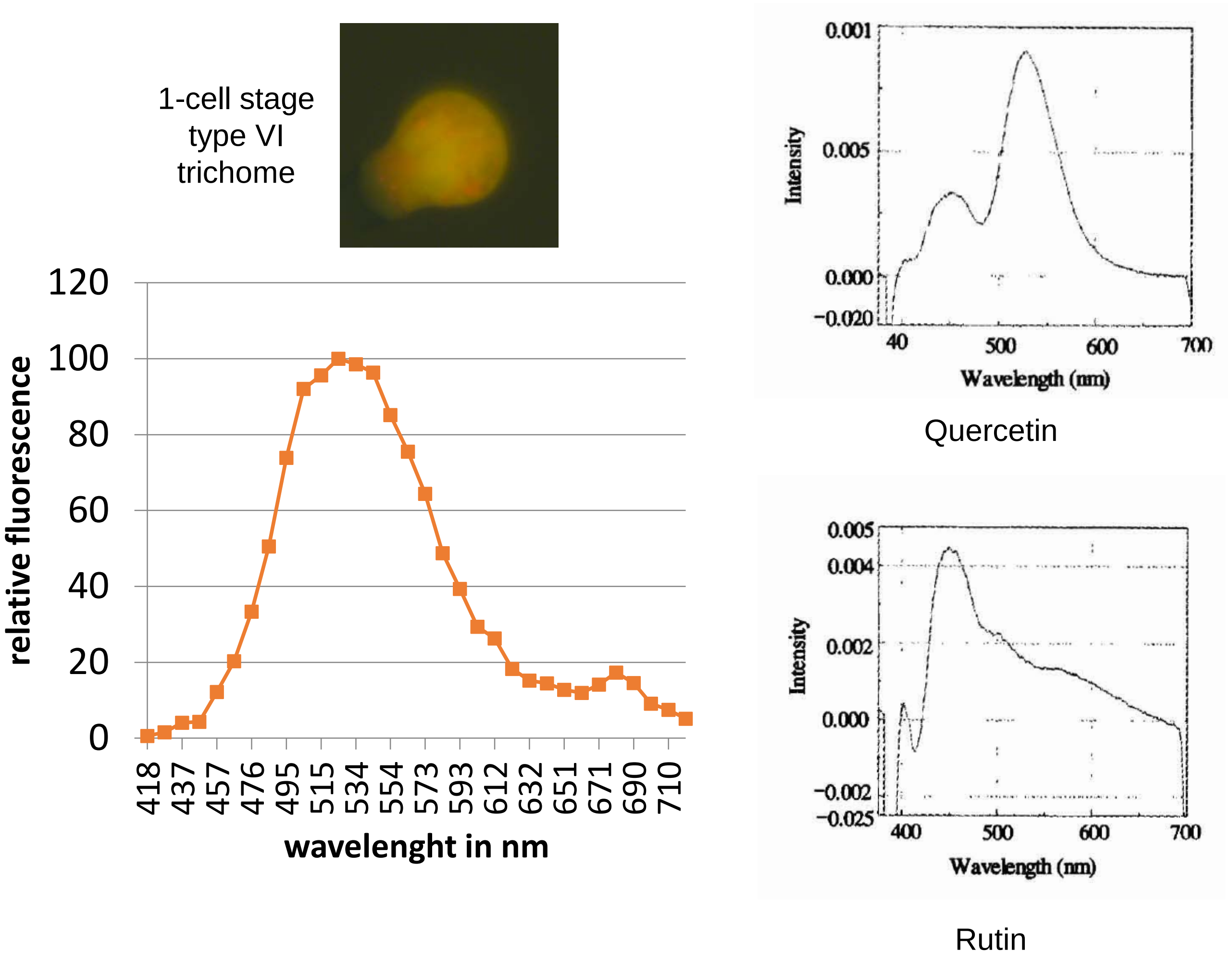

Supplemental Figure 4: Comparison of the emission spectrum of developing type VI trichomes in *S. habrochaites* with that of known flavonoid substances. The emission maximum slightly above 500 nm is more similar to that of quercetin thant to that of rutin.The fluroescene spectra of rutin and quercetin are from Komori *et al.* (2007) Measurement of Rutin and Quercetin in Tartary Buckwheat Flour by Ultraviolet-Induced Fluorescence. *Proceedings of the International Symposium on Buckwheat 2007* pp 403-409.

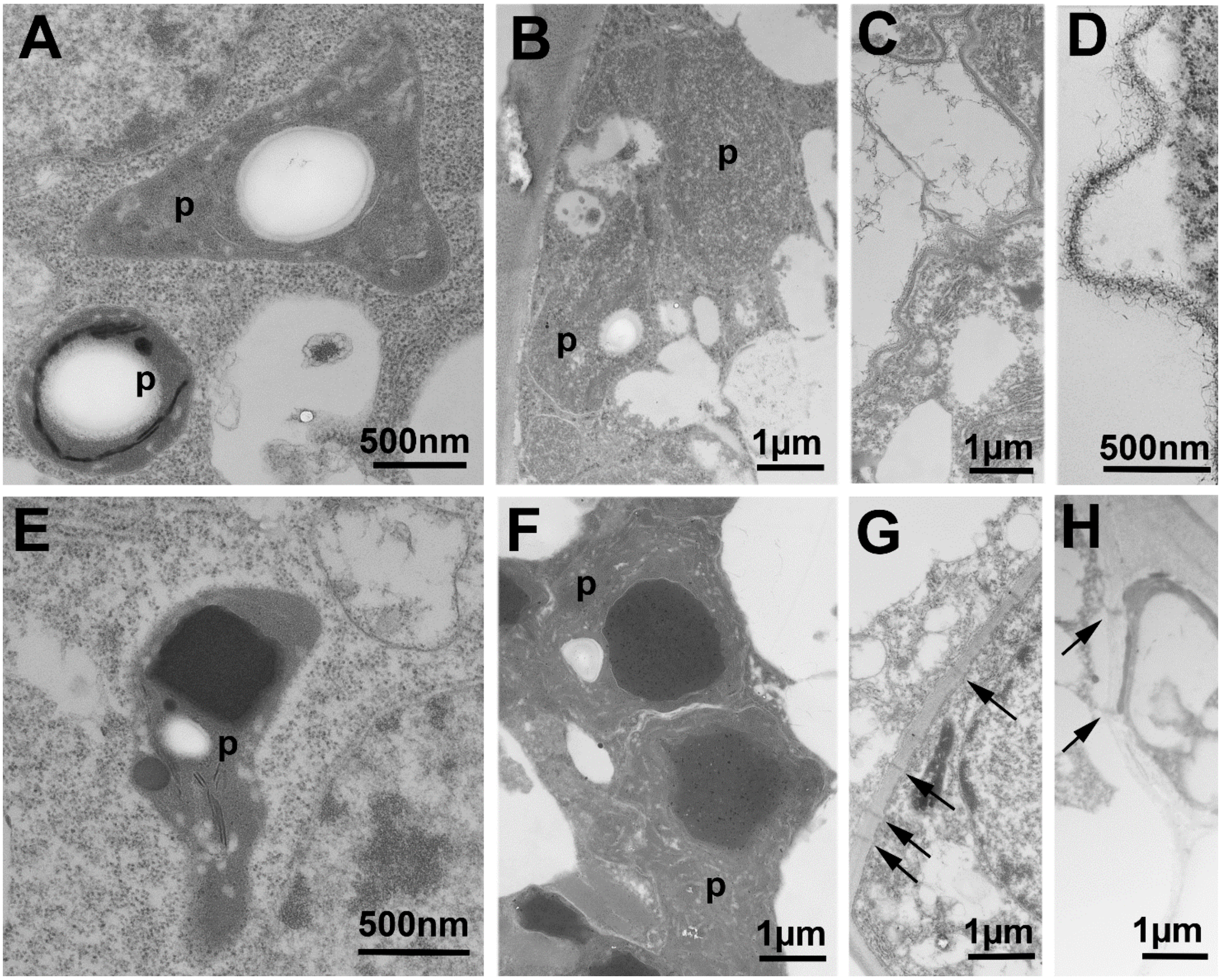

Supplemental Figure 5: Transmission electron microscopy of type VI trichomes in *S. habrochaites* LA1777 (A-D) and *S. lycopersicum* LA4024 (E-F). Details of chloroplasts (p) at the 2-cell stage (A and E). Starch granules are clearly visible. Thylakoids do not build extensive stacks (= grana) as in leaf chloroplasts. At the 4-cell stage (B and F), the thylakoid membranes are hardly visible any more, starch grains can still be seen. Chloroplasts of LA4024 have large electron dense particles (E and F), whereas those are not present in LA1777 in the mature stage (B). C and D: details of the cell wall material during the formation of the intercellular cavity in LA1777. Remains of fibrillous cell wall can be seen. G and H: details of plasmodesmata seen in trichomes of LA4024. G: between the intermediate and the stalk cells. H: between the intermediate and secretory cells. Plasmodesmata are indicated by black arows.

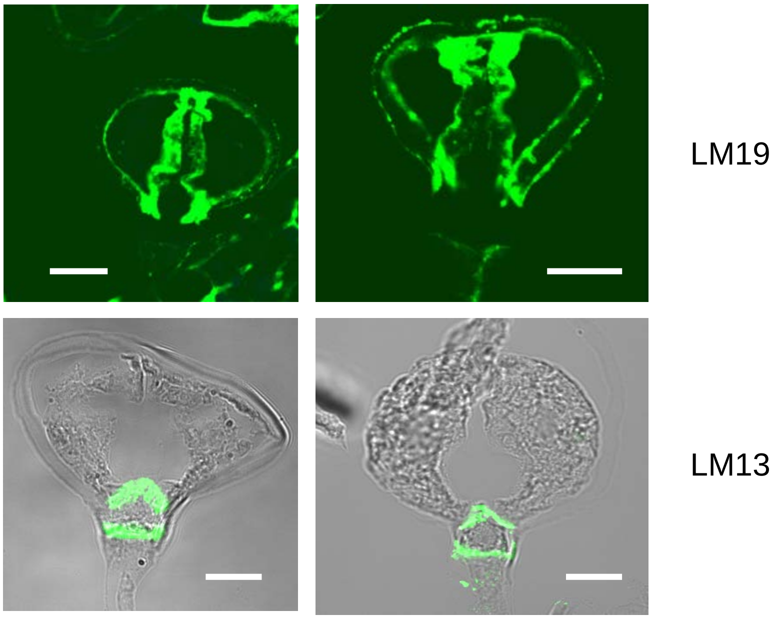

Supplemental Figure 5: Immuno-labelling of type VI trichomes from *S. habrochaites* LA1777. Top row: labelling with LM19, which recognizes homogalacturonan with a preference for unesterified homogalacturonan and LM13 which recognizes linear arabinan. The horizontal bar corresponds to 10 µm.
